# Supplementary material for: The lateralization of left hippocampal CA3 during the retrieval of spatial working memory
Source: Nat Commun. 2020 Jun 9;11:2901. doi: 10.1038/s41467-020-16698-4 (PMC7283476; doi:10.1038/s41467-020-16698-4)
Supplement: Supplementary file 1 — Supplementary Figures [file 41467_2020_16698_MOESM1_ESM.pdf]

Supplementary Data for

The lateralization of left hippocampal CA3 during the retrieval of spatial working memory

Song *et al.*

The supplementary data contains:

Supplementary figure 1
Supplementary figure 2
Supplementary figure 3
Supplementary figure 4
Supplementary table 1

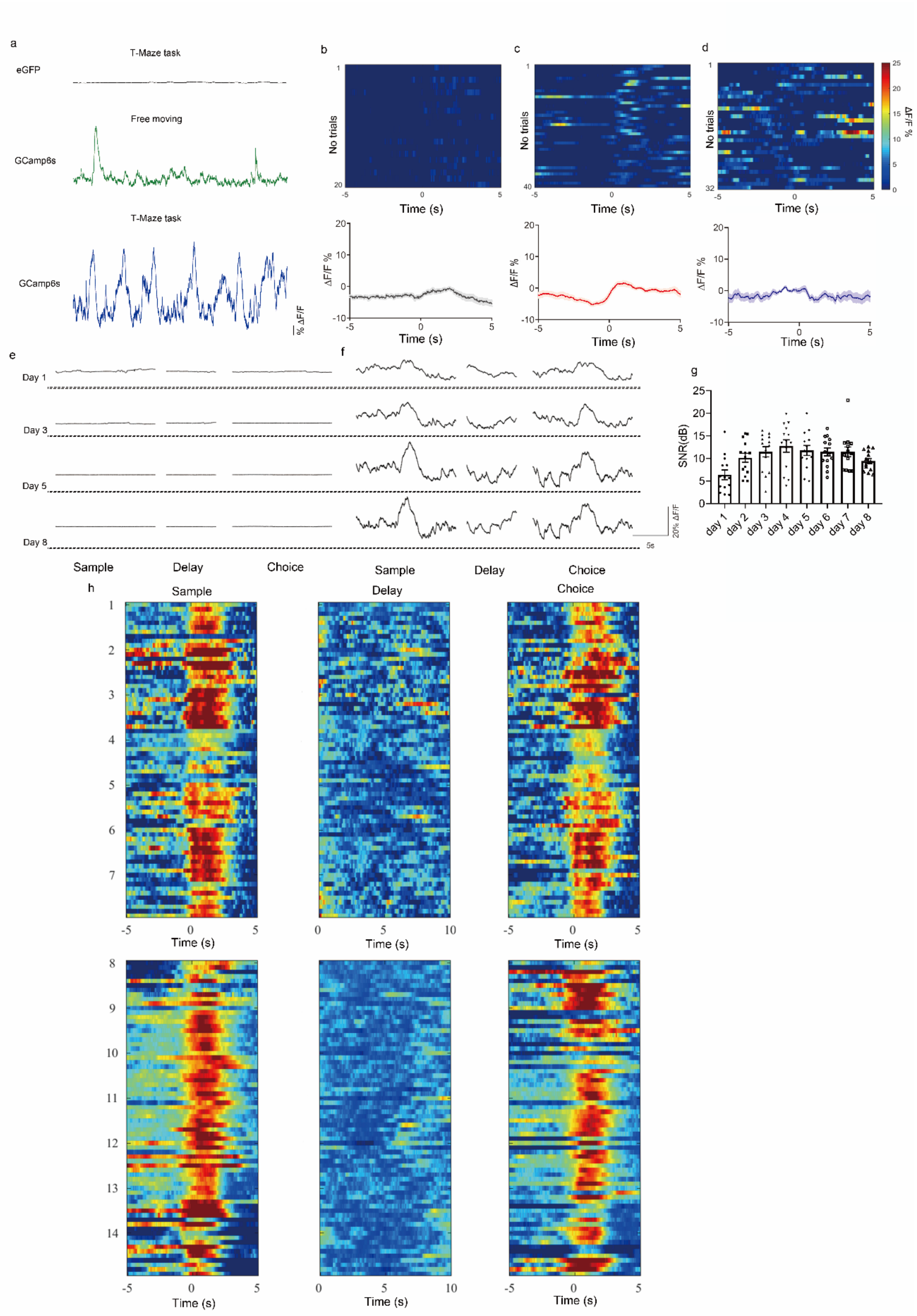

Suppl. Fig. 1 GCaMP6s and eGFP signals in free-moving/T-maze mice. **a** Examples of fluorescent signals expressed by eGFP or GCaMP6s in CA3 during free moving or T-maze tasks. **b** Plot of the mean and SEM of eGFP fluorescence in CA3 of a well-trained mouse at the choice phase and their corresponding heatmap. **c** Plot of the mean and

SEM of Ca^{2+} signal associated with the time points when a free moving animal ran across the location of 'choice begin' and their corresponding heatmap. **d** Plot of the mean and SEM of Ca^{2+} signal associated with the time points when free-running mice were recorded with a barrier (for 10 seconds) at the start point of the main arm, to get reward from each of the goal arm. **e** Long-term eGFP control recording when an example animal did T-maze task on day 1, 3, 5 and 8 during the sample, delay and choice phases. **f** Long-term Ca^{2+} recording when an example animal did T-maze task on day 1, 3, 5 and 8 during the sample, delay and choice phases. **g** The Signal-to-noise ratio (SNR) for the mice that did T-maze task during day 1 to day 8 (n=14 mice); (SNR $= 10 \times \mathrm{Log}10\frac{Calcium\ signal}{GFP\ signal}$). Data are presented as mean ±SEM. **h** Ca^{2+} signal of all recoded mice during the sample, delay and choice phases.

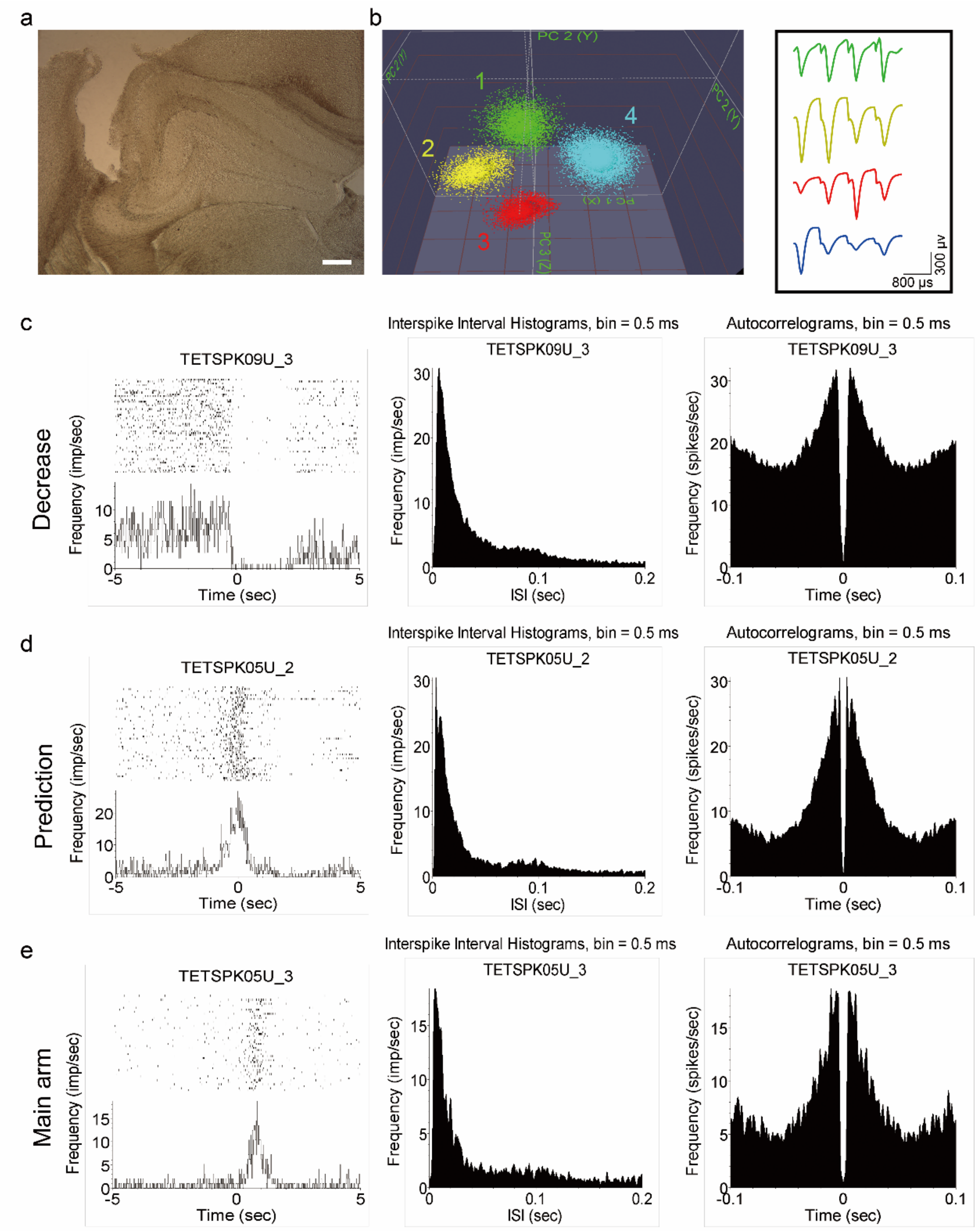

Suppl. Fig. 2 Examples of tetrode recording in CA3. **a** Representative tetrode tracks in the mouse CA3 brain region. Scale bar: 200 µm. **b** Representative four well-isolated units (cluster and waveforms) in the PCA plot recorded from the same tetrode. **c, d, e** Peri-event raster, inter-spike interval histogram and autocorrelogram illustrated example units from decrease (c), prediction (d) and main arm groups (e).

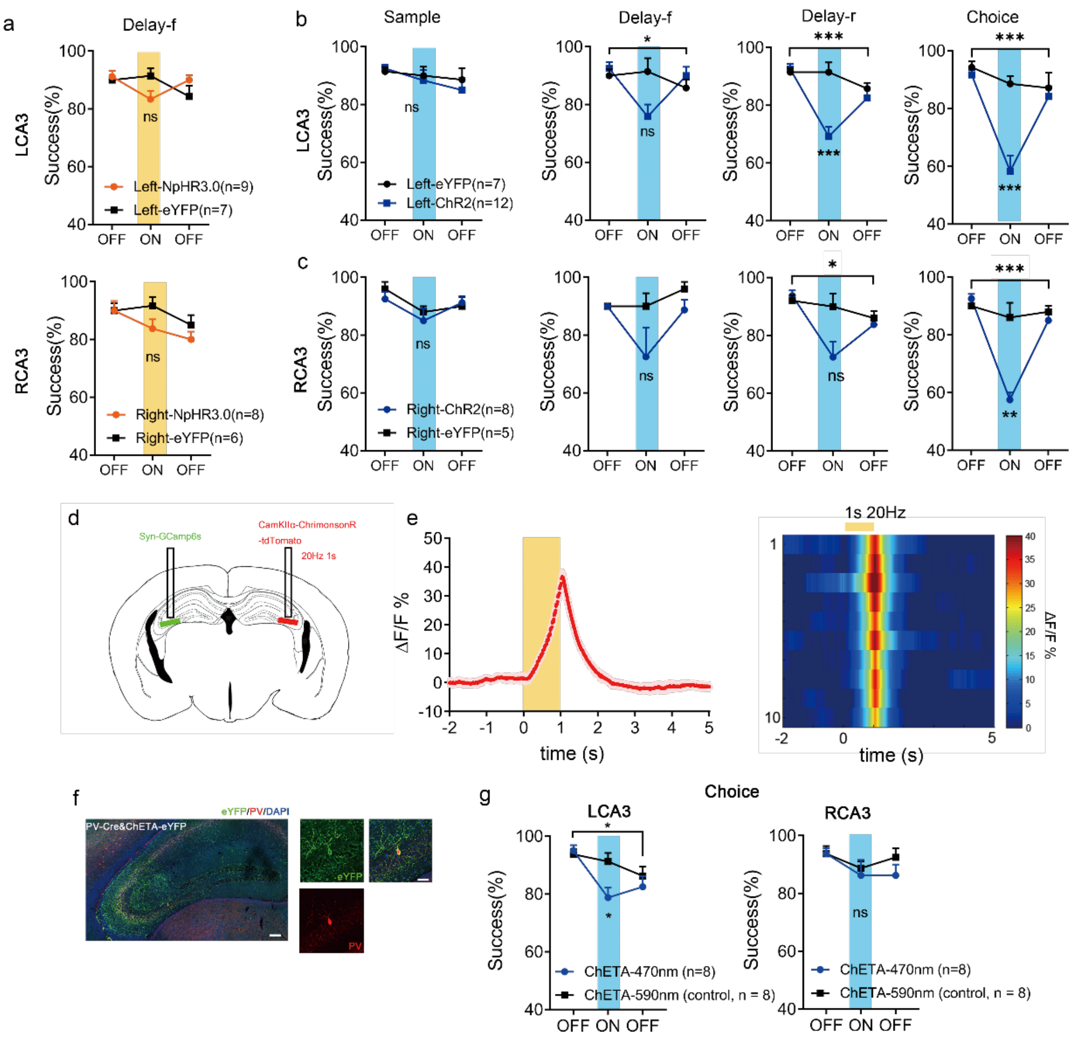

Suppl. Fig. 3 Activation of LCA3/RCA3 neurons in T-maze tasks. **a** Up: percentage of correct performance in mice receiving LCA3 inhibition (n = 7 EYFP (black), n = 9 NpHR3.0 (orange)) during the first 5 seconds of delay phase (rmANOVA, light vs group, P = 0.3476). Down: the percentage of correct performance in mice receiving RCA3 inhibition (n = 6 EYFP, 8 NpHR3.0) during the first 5 seconds of delay phase (rmANOVA, light vs group, P = 0.46). **b** Percentage of correct performance in mice receiving LCA3 activation (n = 7 EYFP (black), n = 12 ChR2 (blue)) during the sample phase (rmANOVA, light vs group, P = 0.77), the first 5 seconds of delay phase (rmANOVA, light vs group, *P = 0.01; multiple comparisons, light on groups, ChR2 vs eYFP, p=0.07), the rest 5 seconds of delay phase (rmANOVA, light vs group, ***P < 0.001), or the choice phase (rmANOVA, light vs group, ***P < 0.001). **c** Percentage of correct performance in mice receiving RCA3 activation (n = 5 EYFP (black), n = 8 ChR2 (blue)) during the sample phase (rmANOVA, light vs group, P = 0.74), during the first 5 seconds of delay phase (rmANOVA, light vs group, P = 0.2751), during the rest 5 seconds of delay phase (rmANOVA, light vs group, *P = 0.04; multiple comparisons, light on groups, p = 0.08), or during the choice phase (rmANOVA, light vs group, ***P < 0.001). **d** Schematic CamkIIα-ChrimonsonR-tdTomato injection in LCA3 and GCamp6s injection in RCA3. **e** Corresponding Ca^{2+} signal in RCA3 region

when activation of LCA3 pyramidal neurons. **f** Confocal images of coronal sections showing ChETA-eYFP expressed neurons (GFP) in CA3 from PV-Cre mice can be co-labeled with PV (red) neurons. Scale bar: left is 200 µm; right is 50 µm. **g** Percentage of correct performance in mice receiving LCA3 (or RCA3) PV neuronal activation (n = 8 ChETA 470nm, n = 8 ChETA 590nm control) during the choice phase (rmANOVA, LCA3: light vs group, *P = 0.0433; RCA3: light vs group, P = 0.6937). Data are presented as mean + SEM (a, b, c, g).

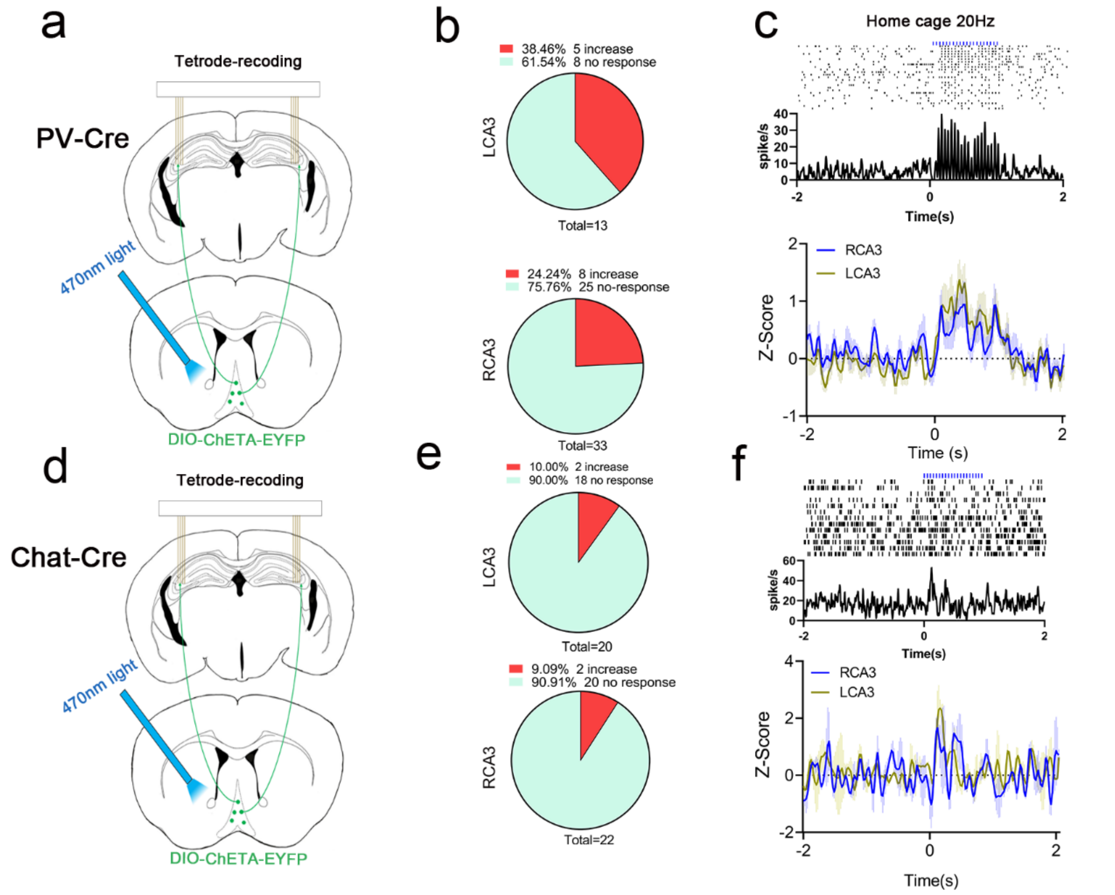

Suppl. Fig. 4 Functional connectivity of $MS^{PV/Chat}$-LCA3/RCA3 pathway. **a** Schematic representation of optogenetic stimulation and *in vivo* recording system. AAV-DIO-ChETA-eYFP was injected into MS of PV-Cre mice and optical fibres were implanted. In addition, 32-tetrodes were placed into bilateral CA3 (n = 4 mice). **b** Subpopulation of LCA3 (up) or RCA3 (down) neurons that were responsive to PV neurons activation of MS through 20Hz stimulation. **c** Up: examples of CA3 neurons that can fire following the optogenetic stimulation of PV neurons in MS. Down: plots of the mean and SEM of Z-score of activated cells that are responsive to MS^{PV} activation in bilateral CA3. **d** Schematic representation of optogenetic stimulation and *in vivo* recording system. AAV-DIO-ChETA-eYFP was injected into MS of Chat-Cre mice and optical fibres were implanted. In addition, 32-tetrodes were placed into bilateral CA3 (n = 3 mice). **e** Subpopulation of LCA3 (up) or RCA3 (down) neurons that were responsive to Chat neurons activation of MS through 20Hz stimulation. **f** Up: examples of CA3

neurons that can fire following the optogenetic stimulation of Chat neurons in MS. Down: plots of the mean and SEM of Z-score of activated cells that were responsive to MS^{Chat} activation in bilateral CA3.

Suppl. Table. 1 Statistic values of animal behavioral tests

Figure	two-way repeated-measures ANOVA (rmANOVA)			Bonferronis multiple comparisons test
	Light vs group	light	group	Light on groups
Fig. 3b Left	P =0.3476<br>F (2, 28) = 1.098	P =0.2806<br>F (2,28) = 1.330	P =0.9103<br>F (1, 14) = 0.01315	P =0.8803<br>t =1.064 ; df =42
Fig. 3b Middle	P =0.21<br>F (2,28) = 1.631	P =0.74<br>F (2, 28) = 0.304	P =0.52<br>(1, 14) = 0.4414	P =0.31<br>t =1.2662 ; df =42
Fig. 3b Right	P =0.0295<br>F (2,28) = 4.008	P =0.0927<br>F (2,28) = 2.593	P =0.0832<br>F (1, 14) = 3.480	P =0.005<br>t =3.359 ; df =42
Fig. 3c Left	P =0.52<br>F (2, 24) = 0.6636	P =0.16<br>F (2, 24) = 1.991	P =0.91<br>F (1, 12) = 0.01357	P> 0.99<br>t =0.4528 ; df =36
Fig. 3c Middle	P =0.54<br>F (2, 24) = 0.635	P =0.46<br>F (2, 24) = 0.793	P =0.90<br>F (1, 12) = 0.01697	P> 0.99<br>t =0.7231 ; df =36
Fig. 3c Right	P =0.97<br>F (2, 24) = 0.03499	P =0.20<br>F (2, 24) = 1.714	P =0.60<br>F (1, 12) = 0.2911	P> 0.99<br>t =0.4262 ; df =36
Fig. 4b-Left	P =0.0146<br>F(2, 28) = 4.932	P =0.0949<br>F (2, 28) = 2.565	P =0.0096<br>F (1, 14) = 8.992	P =0.0006<br>t =4.1 ; df =42
Fig. 4b-Right	P =0.2092<br>F (2, 24) = 1.671	P =0.1584<br>F (2, 24) =1.992	P =0.7148<br>F (1, 12) = 0.14	P =0.6648<br>t =1.244 ; df =36
Supplementary Fig. 3a Up	P =0.03<br>F (2,28) = 3.853	P =0.34<br>F (2, 28) = 1.132	P =0.82<br>F (1, 14) = 0.05578	P =0.07<br>t =2.36 ; df =42
Supplementary Fig. 3a Down	P =0.46<br>F (2, 24) = 0.8008	P =0.07<br>F (2, 24) = 2.952	P =0.11<br>F (1, 12) = 2.958	P =0.25<br>t =1.788 ; df =36

Supplementary Fig.	P =0.77	P =0.29,	P =0.67	P> 0.99
3b-1	F (2,34) = 0.2586	F (2, 34) = 1.276	F (1, 17) = 0.1893	t =0.3386 ; df =51
Supplementary Fig.	P =0.01	P =0.11	P =0.33	P =0.009
3b-2	F (2, 34) = 4.954	F (2, 34) = 2.387	F (1, 17) = 1.022	t =3.137 ; df =51
Supplementary Fig.	P < 0.001	P =0.002	P =0.01	P< 0.001
3b-3	F (2,34) = 8.614	F (2, 34) = 7.905	F (1, 17) = 8.324	t =4.992 ; df =51
Supplementary Fig.	P < 0.001	P < 0.001	P =0.005	P< 0.001
3b-4	F (2,34) = 8.587	F (2, 34) = 13.31	(1, 17) = 10.61	t =5.269 ; df =51
Supplementary Fig.	P =0.74	P =0.09	P =0.46	P> 0.99
3c-1	F (2, 22) = 0.3071	F (2, 22) = 2.711	F (1, 11) = 0.5803	t =0.6697 ; df =33
Supplementary Fig.	P =0.2751	P =0.1403	P =0.1657	P =0.1264
3c-2	F (2, 22) = 1.369	F (2, 22) = 2.432	F (1, 11) = 2.204	t =2.114 ; df =33
Supplementary Fig.	P =0.04	P =0.01	P =0.07	P =0.006
3c-3	F (2, 22) = 3.813	F (2, 22) = 5.230	F (1, 11) = 4.092	t =3.378 ; df =33
Supplementary Fig.	P < 0.001	P < 0.001	P =0.006	P<0.001
3c-4	F (2, 22) = 17.47	F (2, 22) = 26.41	F (1, 11) = 11.30	t =6.588 ; df =33
Supplementary Fig.	P =0.0433	P =0.0009	P =0.1005	P =0.0133
3g-Left	F (2, 28) = 3.519	F (2, 28) = 9.119	F (1, 14) = 3.092	t =3.009 ; df =42
Supplementary Fig.	P =0.6937	P =0.2319	P =0.2016	P>0.9999
3g-Right	F (2, 28) = 0.3705	F (2, 28) = 1.540	F (1, 14) = 1.796	t =0.5265 ; df =42
	**two-tailed unpaired Mann-Whitney-U-test**			
Fig. 1c-Right	p = 0.0131			
Fig. 1d-Right	p = 0.3447			
Fig. 3e-Right	P = 0.4135			
Fig. 3f-Right	P = 0.0297			
	**two-tailed paired t-test**			

Fig. 4d-Left-LCA3	P =0.1394; t =1.703; df = 6
Fig. 4d-Left-RCA3	P =0.6548; t =0.4822; df = 4
Fig. 4d-Right-LCA3	P =0.0033; t =5.248; df = 5
Fig. 4d-Right-RCA3	P =0.5520; t =0.6486; df = 4
Fig. 6c-LCA3	P =0.0019; t =5.284; df = 6
Fig. 6c-RCA3	P >0.9999; t =0; df = 4
Fig. 6f-LCA3	P =0.2031; t =1.464; df = 5
Fig. 6f-RCA3	P =0.7412; t =0.3492; df = 5
